# *Aspergillus fumigatus* establishes infection in zebrafish by germination of phagocytized conidia, while *Aspergillus niger* relies on extracellular germination

**DOI:** 10.1038/s41598-019-49284-w

**Published:** 2019-09-05

**Authors:** Bjørn E. V. Koch, Natalia H. Hajdamowicz, Ellen Lagendijk, Arthur F. J. Ram, Annemarie H. Meijer

**Affiliations:** Institute of Biology Leiden, Leiden University, Leiden, The Netherlands

**Keywords:** Eukaryote, Antimicrobial responses, Fungal immune evasion

## Abstract

Among opportunistically pathogenic filamentous fungi of the *Aspergillus* genus, *Aspergillus fumigatus* stands out as a drastically more prevalent cause of infection than others. Utilizing the zebrafish embryo model, we applied a combination of non-invasive real-time imaging and genetic approaches to compare the infectious development of *A*. *fumigatus* with that of the less pathogenic *A*. *niger*. We found that both species evoke similar immune cell migratory responses, but *A*. *fumigatus* is more efficiently phagocytized than *A*. *niger*. Though efficiently phagocytized, *A*. *fumigatus* conidia retains the ability to germinate and form hyphae from inside macrophages leading to serious infection even at relatively low infectious burdens. By contrast, *A*. *niger* appears to rely on extracellular germination, and rapid hyphal growth to establish infection. Despite these differences in the mechanism of infection between the species, galactofuranose mutant strains of both *A*. *fumigatus* and *A*. *niger* display attenuated pathogenesis. However, deficiency in this cell wall component has a stronger impact on *A*. *niger*, which is dependent on rapid extracellular hyphal growth. In conclusion, we uncover differences in the interaction of the two fungal species with innate immune cells, noticeable from very early stages of infection, which drive a divergence in their route to establishing infections.

## Introduction

The *Aspergillus* genus comprises over 200 species of filamentous fungi. Though normally harmless, several *Aspergillus* species have been described as opportunistic pathogens, capable of causing invasive aspergillosis, a severe condition characterized by the germination of spores, growth and penetration of fungal hyphae into host tissues. *A*. *fumigatus* is the dominating causative agent in invasive aspergillosis with four species (*A*. *niger*, *A*. *flavus*, *A*. *tereus* and *A*. *nidulans*) accounting for almost all remaining incidences^1^. Several different features of *A*. *fumigatus* physiology have been proposed to account for its higher virulence, including the size of the conidia, their ubiquitous abundance and the hydrophobicity of the *A*. *fumigatus* cell wall making the spreading of their conidia highly efficient^2,3^. In addition to these features, however, it seems clear that different pathogenic species of *Aspergillus* may interact with components of the host innate immune system in fundamentally different ways which may be another important factor in the increased pathogenicity of *a*. *A*. *fumigatus* compared to other *Aspergillus* species^4^. For this reason, thorough characterization of the interactions between host and pathogen, as well as the identification of common factors that may influence virulence in similar ways across species, is of interest as potential targets for therapeutic interventions.

The fungal cell wall is essential to the interactions between the fungus and the host immune system. The fungal cell wall is a protective structure consisting of a complex mixture of various polysaccharides and glycoproteins. The outer layer of the cell wall is enriched in glycoproteins which are retained in the cell wall by ionic interactions or by covalent linkage via remnants of GPI-anchors to the glycan/chitin part of the cell wall. The inner layer of the cell wall consists of mainly of β-1,3-glucan and chitin. These structural components are cross-linked to each other and form an important scaffold to which other cell wall components (α-glucan, galactomannan and glycoproteins) can bind^5,6^. Galactofuranose (Gal*f*) containing glycostructures (mainly galactomannan and galactomannoproteins) are common components of *Aspergillus* cell walls^7^ and Gal*f* biosynthesis is important to maintain cell wall integrity. Mutations or deletions of genes required for Gal*f-*biosynthesis lead to higher sensitivity towards antifungal drugs^8–10^. UDP-galactopyranose mutase is a crucial enzyme in the biosynthesis of Gal*f-*containing glycoconjugates. The enzyme is required to convert UDP-galactopyranose into UDP-galactofuranose which is used as a nucleotide sugar donor for the transfer of Gal*f* via Golgi localized Gal*f*-transferases^11^. The gene encoding UDP-galactopyranose mutase (named *ugmA* in *A*. *niger* and *A*. *nidulans*, and *glfA* in *A*. *fumigatus*) has been shown to be required for Gal*f* biosynthesis^8,9,12,13^.

From a pharmaceutical perspective, Gal*f-*containing glycostructures are interesting for two reasons. First, the presence or absence of Gal*f* in *A*. *fumigatus* has been shown to influence virulence and resistance to antifungal drugs in immunosuppressed mice, making it an interesting target either on its own or in combination with other antifungal agents^9^. Second, while the six-membered pyranosyl form of the hexose monosaccharide galactose is found in many vertebrate species as part of complex glycoconjugates of proteins and lipids^14^, the five-membered furanosyl form is not found in higher vertebrates^7^, thus making it a candidate for selective pharmaceutical intervention. Immunogenic properties of Gal*f* have been shown *in vitro*^15^, but the importance of Gal*f* as a pathogen-associated molecular pattern (PAMP) has been contested *in vivo*^16^. Further studies into the effects of mutations of genes involved in the biosynthesis of Gal*f* in relation to infectious behavior are required to establish the role of Gal*f* and derived glycosides in pathogenic fungal infection.

The zebrafish embryo is an attractive model for *in vivo* studies of *Aspergillus* infection. The optical transparency of the embryo makes it very tractable for fluorescence based live *in vivo* studies of the development of fungal morphology and the interactions of the fungal pathogen with innate immune cells. While the embryo possesses a functional innate immune system with macrophages and neutrophils capable of phagocytizing microbes as early as 30 hours post fertilization^17,18^, the first mature lymphocytes do not appear until 3 weeks post fertilization with the progressing development of the pronephronic tissues from which these cells primarily originate^19–21^. This temporal gap between the emergence of components of innate and acquired immunity allows for studying opportunistic infections without the need for immunosuppression of the host. The feasibility of this concept has recently been demonstrated by infections with *A*. *fumigatus*^22^, *Mucor circinelloides*^23^ and *Cryptococcus neoformans*^23,24^, and as the hindbrain ventricle is devoid of leukocytes at the early stages of embryonic development^17^ it has been widely utilized to study leukocyte migratory patterns.

Here we utilize the zebrafish embryonic hindbrain injection model to characterize the disease progression resulting from *A*. *fumigatus* and *A*. *niger* conidia injections in terms of immune cell interactions and embryonic survival, and compare the results to those of the Gal*f*-deficient mutants. We find that *A*. *niger* and *A*. *fumigatus* elicit similar immune responses, but interact with innate immune cell populations differently, causing the two *Aspergillus* species to follow diverging paths of pathogenic development. Whereas *A*. *niger* appears dependent on rapid growth to overwhelm the innate immune response, *A*. *fumigatus* is able to germinate and grow after the conidia have been phagocytized by macrophages. Yet, in spite of the clear differences in infectious development, the Gal*f*-deficient strains of both species are significantly attenuated in pathogenicity. The impeded virulence of the Gal*f-*deficient strains appears to correlate most strongly with decreased hyphal growth rate. Therefore, in agreement with the notion that *A*. *niger* proliferation is more dependent on speed of growth, we find the effect of Gal*f* deficiency to be more drastic in this species. These results demonstrate the value of the zebrafish embryo model for comparative *in vivo* studies of *Aspergillus* infection and evaluating the impact of cell wall components.

## Results

### *Aspergillus niger* and *Aspergillus fumigatus* exhibit differences in infectious behavior that are related to their interactions with host innate immune cells

**Figure 1:**
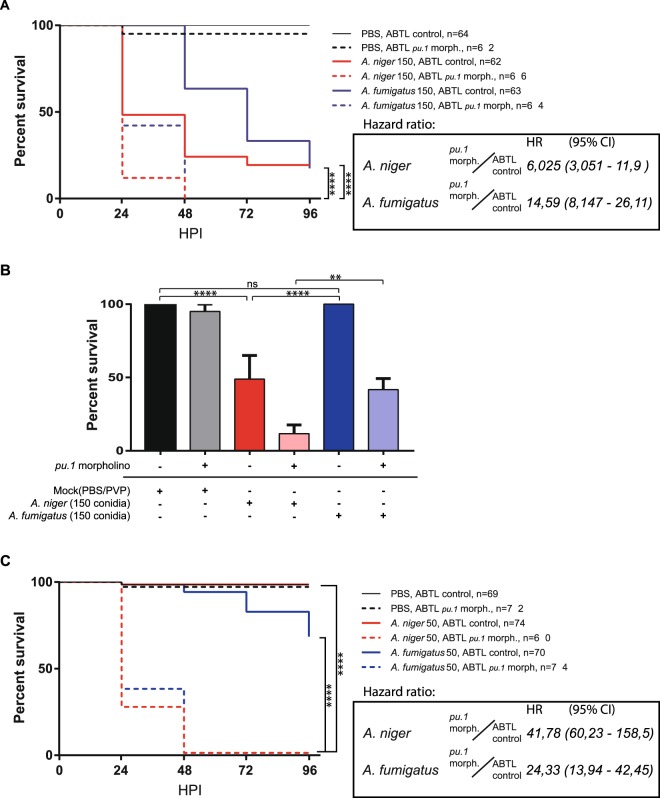
Survival rate analysis of *A*. *fumigatus* and *A*. *niger* infection reveals a difference in pathological development dependent on the presence of innate immune cells. (**A**) Survival of control (no morpholino) and immunodeficient *pu*.*1* morphant zebrafish embryos infected with 150 conidia of *A*. *fumigatus* and *A*. *niger* at 28 HPF, survival was monitored at 24, 48, 72 and 96 hours post infection (HPI). Mean survival 24 HPI (*A*. *niger*), 48 HPI (*A*. *fumigatus*). (**B**) Temporal specific statistical analysis of survival of control and immunodeficient *pu*.*1* morphant zebrafish embryos infected with 150 conidia of *A*. *fumigatus* and *A*. *niger* monitored at 24 HPI, based on the data presented in (**A**). (**C**) Survival of control (no morpholino) versus *pu*.*1* morpholino injected zebrafish embryos infected 50 conidia of *A*. *fumigatus* and *A*. *niger*. Each graph displays combined data from three independent biological replicates. (**A**,**C**) Statistical curve comparisons by Mantel-Cox test, ****p ≤ 0,0001. Hazard ratios calculated by Mantel-Haenszel method. (**B)** **p ≤ 0,01; ****p ≤ 0,0001 by 2-way anova with Turkey’s multiple comparisons test.

To investigate the pathogenic behavior of *A*. *niger* and *A*. *fumigatus* we applied the approach of Knox *et al*.^22^ for hindbrain ventricle injection of fungal conidia in zebrafish embryos. Forming a cavity lined with epithelium, the hindbrain ventricle in zebrafish embryos is commonly used to mimic the conditions of alveolar infections^24–26^. This is an infection route that we and others have successfully used to study macrophage and neutrophil responses to bacterial infections^25–27^. Using this approach, we investigated the survival of control embryos versus *pu*.*1* morpholino injected embryos after infection with *A*. *niger* and *A*. *fumigatus*. Morpholino knockdown of *pu*.*1* causes a blockage of myelopoiesis, the process by which the macrophages and neutrophils develop^28^, thus rendering the *pu*.*1* morphant embryo deficient in cellular innate immune responses for the duration of the experiment. Our observations revealed that *A*. *niger* and *A*. *fumigatus* both exhibited robust pathogenicity over 96 hours in survival rate assays at high infectious burdens of 150 conidia (Fig. 1A), and in both cases the innate immune system appeared to confer significant protection, with a hazard ratio of approximately 6 between controls and *pu*.*1* morphants for *A*. *niger* and over 14 for *A*. *fumigatus*. However, though overall mortality rates to these high infectious burdens were similar at the termination of the experiment, 96 hours post infection (HPI), there was a marked difference in the temporal development of survival rate assays between the two *Aspergillus* species. While the profile of *A*. *niger* was characterized by significant mortality at 24 HPI, the sharpest drop in survival in the case of *A*. *fumigatus* occurred at later time points. This indicates that the development of infection by the two different fungal pathogens is different, causing mortality to occur later in *A*. *fumigatus* infection (Fig. 1A,B). Decreasing the fungal challenge dose revealed that the mortality of *A*. *niger* was more dependent on high infectious burdens than *A*. *fumigatus*, as *A*. *niger* caused no mortality with a challenge dose of 50 conidia, while *A*. *fumigatus* still induced significant mortality at this challenge dose (Fig. 1C). This is consistent with the general view that *A*. *fumigatus* is more pathogenic than *A*. *niger*. The protective effect of the host immune system was once again demonstrated as both *A*. *niger* and *A*. *fumigatus* induced nearly complete mortality in immunodeficient *pu*.*1* morphants. The protective effect, as quantified by the hazard ratios between control and *pu*.*1* morphants confirm this observation; while *pu*.*1* morphants are 24 times more likely to die from dose of 50 conidia of *A*. *fumigatus* over 96 hours than control embryos, that ratio is nearly 42 for *A*. *niger*. Thus, it appears that the interactions of the injected fungal conidia with the innate immune system shapes the outcome of the infection, and that the innate immune system confers a greater protection against a low challenge dose of *A*. *niger* than against *A*. *fumigatus*. As we wished to investigate the germination and hyphal growth phases of infectious development we continued with the higher challenge doses of 150 conidia in the subsequent experiments.

In summary, these survival rate analyses indicated that *A*. *niger* and *A*. *fumigatus* both exhibit robust pathogenicity when high infectious burdens of 150 conidia are applied, but that there is a temporal difference in the infectious development, whereby *A*. *niger* induces robust mortality within the first 24 hours, while *A*. *fumigatus* takes longer to deliver a lethal outcome. The innate immune system confers significant protection against both species of *Aspergillus*, but while the innate immune system is more protective against *A*. *fumigatus* than *A*. *niger* at high challenge doses, this is reversed at lower challenge doses. All these observations led us to conclude that the two *Aspergillus* species develop infection through different routes, and that the differences are likely related to interactions with the innate host immune system.

### Both species induce similar leukocyte migratory responses but *Aspergillus fumigatus* is more efficiently phagocytized than *Aspergillus niger*

**Figure 2:**
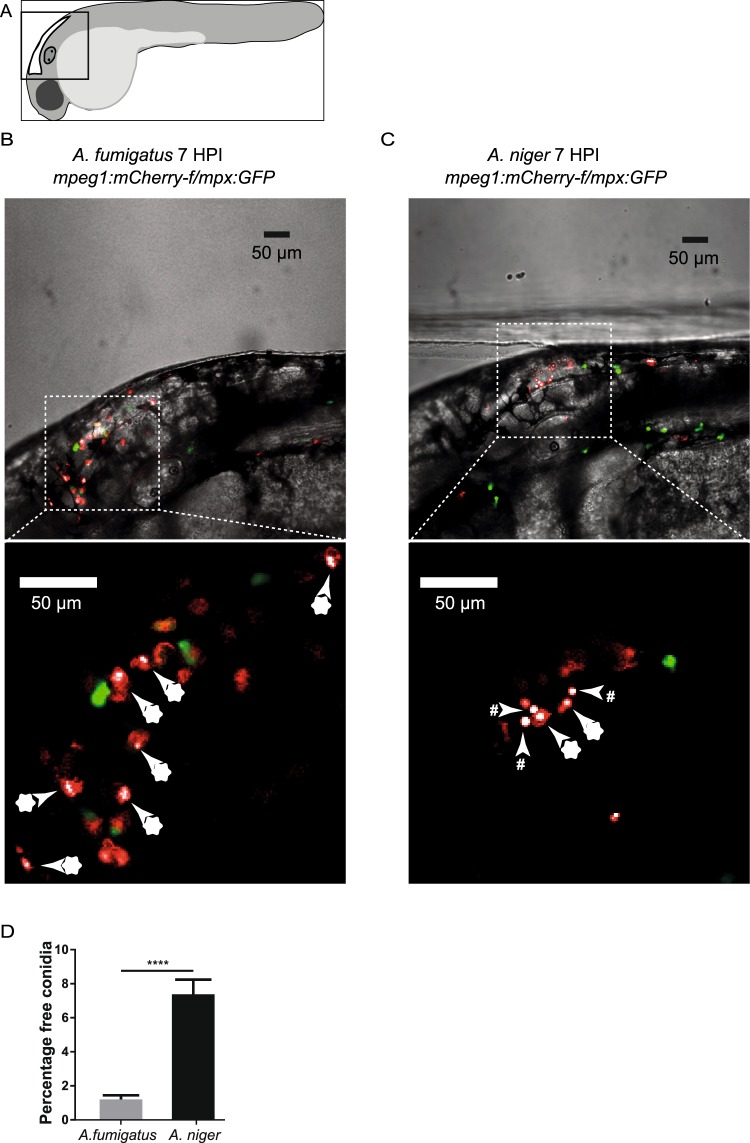
Differences in phagocytic efficiency of *A*. *fumigatus* and *A*. *niger* conudia at 7 hours post infection. (**A**) Schematic drawing of the embryo and the area imaged in the figure. (**B**,**C**) Single confocal microscopy planes of the hindbrain of zebrafish embryos injected with 150 conidia of *A*. *fumigatus* (**B**) and *A*. *niger* (**C**) at 7 hours post infection (HPI). Macrophages and neutrophils are labelled with farnesylated - membrane bound - mCherry and cytosolic eGFP respectively, and *Aspergillus* conidia with non membrane bound mCherry. Blow-up images without brightfield are shown below to illustrate the different appearance of phagocytosed (marked with asterisks) and free conidia (marked with hashtags). (**D**) Quantification of the percentage of the challenge dose left unphagocytized at 7 HPI in *A*. *fumigatus* (1,2%) versus *A*. *niger* (7,3%). Bars represent mean ± s.e.m. combined from 3 biological replicates, n = 40–47, ****p ≤ 0,0001 by t-test with Welch’s correction.

**Figure 3:**
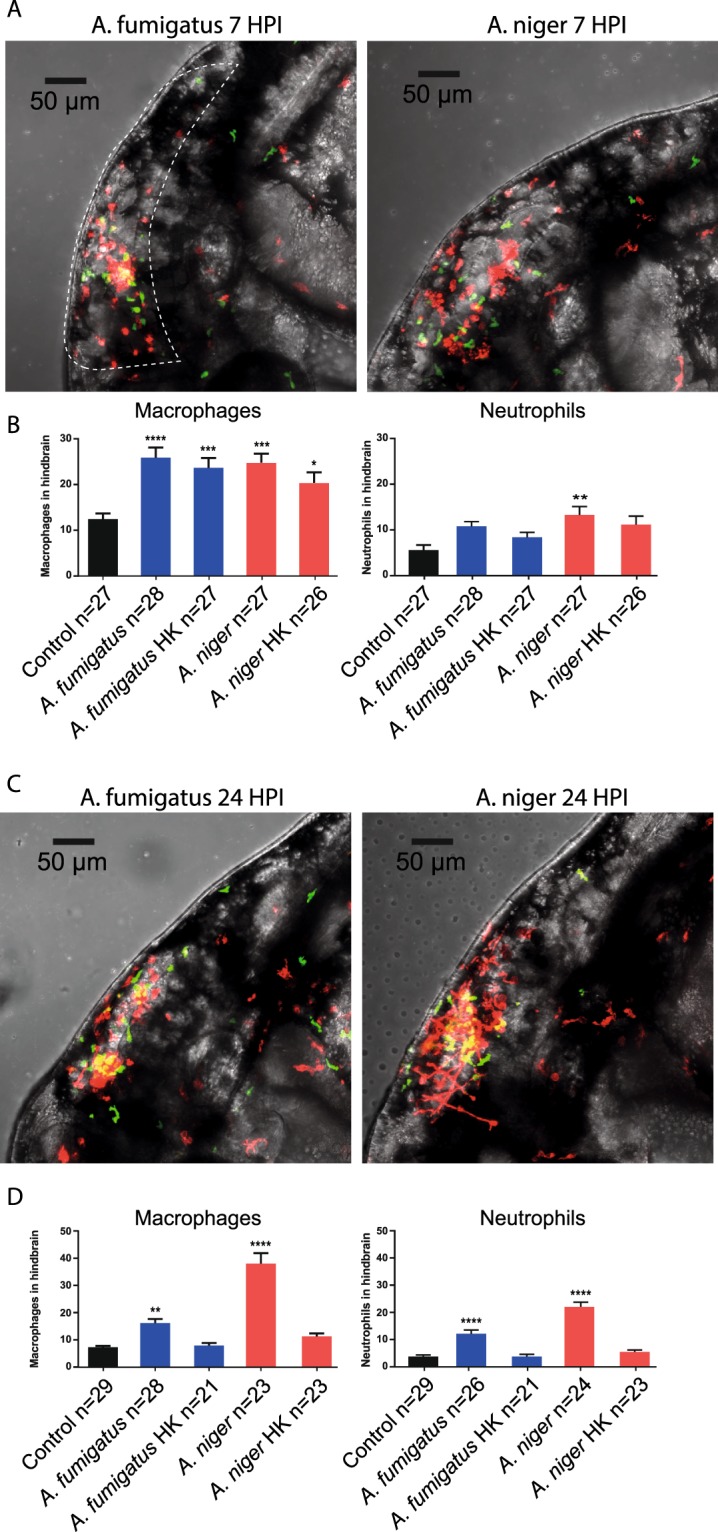
Quantification of leukocyte migration to the hindbrain at early and later stages of infection. (**A**,**C**) Representative confocal stacks of Tg (*mpeg1*:*mCherry*; *mpx*:*GFP*) zebrafish embryos with macrophages and neutrophils labelled in red and green respectively, injected with 150 conidia of *A*. *fumigatus* and *A*. *niger* at 7 HPI (**A**) and 24 HPI. (**C**) Stacks formed the basis of quantification of leukocyte migration to the hindbrain. As Macrophages and conidia were labelled with the same fluorophore, intensity of signal and morphology was used to distinguish conidia from macrophages. (**B**,**D**) Quantification of leukocyte migration in the hindbrain of embryos injected with *A*. *fumigatus* or *A*. *niger*, live or heat-killed (HK) conidia, compared to control injections (2% PVP/PBS) at 7 HPI (**B**) and 24 HPI. (**D**) *p ≤ 0,05; **p ≤ 0,01; ***p ≤ 0,001; ****p ≤ 0,0001 by one-way ANOVA with Dunnett’s multiple comparisons test, n = 21–30 per group from 3 biological replications. The quantifications represent a part of a larger data-set which is presented in full in Supplemental Fig. 1. The statistical analysis presented is based on the full dataset.

We wished to investigate whether the apparent difference in the infectious progression between *A*. *niger* and *A*. *fumigatus* was caused by differences in their interactions with the different subsets of innate immune cells. To approach this question, we characterized the nature of these interactions in the hours immediately subsequent to infection. To this end, we utilized a dual reporter zebrafish line with labelled neutrophils^29^ and macrophages^30^ in green and red, respectively. As the mCherry signal labelling the macrophages in this line is membrane bound, we were able to use morphology and brightness of fluorescent signal to distinguish between macrophages and fungi (Fig. 2B,C). Examining confocal slices carefully we were able to establish a difference in the efficiency of phagocytosis between the two *Aspergillus* species, as early as 7 hours post infection (HPI), before germination of the conidia. We found that *A*. *fumigatus* was significantly more efficiently phagocytized than *A*. *niger*. Overall, on average 7,3% of the conidia of *A*. *niger* were free and unphagocytized, at 7 HPI, against just 1,2% unphagocytized *A*. *fumigatus* conidia (Fig. 2D). Using the same approach we investigated the migratory behavior of each leukocyte population. Again we relied on morphology and brightness of fluorescent signal to distinguish between macrophages and fungi (Fig. 3). To investigate the development over time and assess the importance of fungal lifecycle dynamics we included live and heat killed conidia, at 7 and 24 HPI. The quantification results indicated quite similar leukocyte migratory behavior directed against both species of *Aspergillus*. Heat killed conidia of either species elicited a significant macrophage migratory response indicating that the initial macrophage response is likely to be independent of the maturation and swelling of the conidia prior to germination (Fig. 3). However, while the leukocyte presence in response to live conidia of both species were significantly elevated at 24 HPI the leukocyte numbers in subjects injected with heat killed conidia of both species had reverted to the level of control injected subjects. Together with the survival rate observations this indicates that the efficient phagocytosis of injected *A*. *fumigatus* is not sufficient to resolve the infection, and that significant neutrophil influx occurs later than is the case of *A*. *niger*.

### *Aspergillus niger* often germinate and grows hyphae extracellularly while *Aspergillus fumigatus* conidia persist, germinate, and grow from inside macrophages

**Figure 4:**
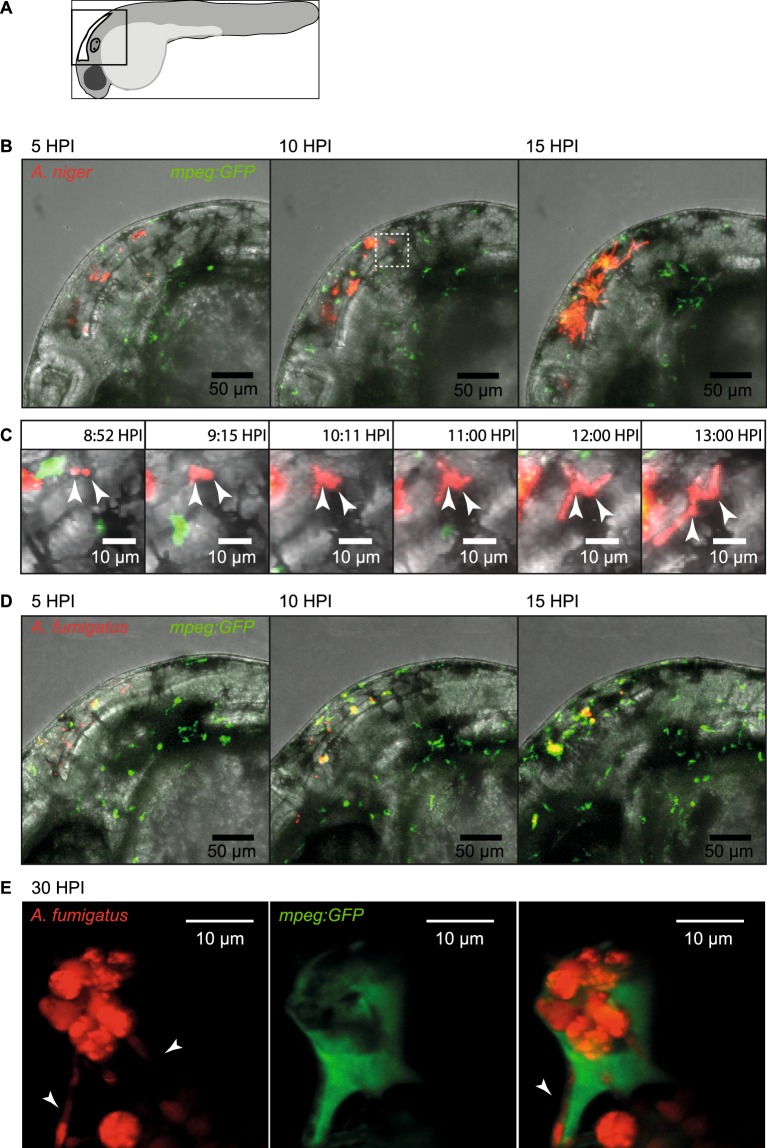
Confocal microscopy captures different infectious development of *A*. *niger* and *A*. *fumigatus*. (**A**) Schematic drawing of the embryo and the area imaged in the figure. (**B**) Still images from confocal time-lapse microscopy (Supplementary Movie SM1), depicting the typically rapid development of *A*. *niger* infection. Images from 5, 10 and 15 HPI of 150 conidia of *A*. *niger* in Tg(*mpeg1:EGFP*). At 15 HPI several conidia has germinated and progressive hyphal growth is evident. (**C**) Digitally magnified still images from Supplementary Movie SM1, aiming to capture the events of extracellular germination and hyphal growth of two separate conidia from approximately 9 HPI to 13 HPI. (**D**) Still images from confocal time-lapse microscopy (Supplementary Movie SM3), depicting the development of *A*. *fumigatus* infection at 5, 10 and 15 HPI, after injection of 150 conidia of *A*. *fumigatus* in Tg(*mpeg1:EGFP*). No germination events were detectable at the end of the time-lapse (Supplementary Movie SM3). (**E**) High magnification confocal microscopy showing a cluster of *A*. *fumigatus* conidia in the hindbrain of a Tg(*mpeg1:EGFP*) zebrafish embryo acquired at 30 hours post infection of 150 conidia. In the left panel, showing only the red fluorescence channel, several hyphae could be seen protruding from the cluster of several conidia (arrowheads). In the central panel, showing the green channel the macrophage can be seen. In the right panel, overlaying the green fluorescence channel it was clear that the cluster of conidia had been phagocytized and were contained within a macrophage. One growing hyphae could be seen to stretch the membrane of the macrophage (arrowhead). (**B**) White box insert in 10 HPI image represents the magnified area in image panel (C).

Following the development in confocal time lapse experiments (between 4 and 24 HPI), the initial differences in infectious behavior remained evident and were further perpetuated as the conidia germinated and hyphal growth commenced. In *A*. *niger* infection, in every instance when germination was observed, it occurred from non-phagocytized conidia with germination and hyphal growth commencing as early as 9–12 HPI (more than 100 observations of germination events from embryos injected in 3 independent experiments) (see Supplementary Movies SM1, SM14 and SM15). The outcome, in terms of larval survival appeared to be dependent on the success of immune cells in the ensuing, highly inflammatory response, to attenuate the growth of the hyphae (Fig. 4B,C and Supplementary Movies SM1, SM2, SM14 and SM15). The more rapid development observed in *A*. *niger* infection appears in good agreement with our initial observations that *A*. *niger* caused mortality more rapidly than *A*. *fumigatus* (Fig. 1).

Since *A*. *fumigatus* conidia were efficiently phagocytized by macrophages in the hours immediately after injection, it stands to reason that the development of infection and pathogenicity would be different from that of *A*. *niger*. Indeed, in the cases analyzed, *A*. *fumigatus* germination and hyphal growth occurred later than in the case of *A*. *niger*, (compare Fig. 4B,D) commencing around 24–48 HPI (see Supplementary Movie SM5). Confocal microscopy revealed that, at least in some cases, *A*. *fumigatus* hyphal growth occurred from conidia which had been phagocytized by macrophages (Fig. 4E). This was an event we had not observed in *A*. *niger* infection, though we cannot rule out that it may happen occasionally. With the aim to capture the event of germination and hyphal growth from macrophages after phagocytosis in a time lapse movie, and to further clarify the role of neutrophils and macrophages in the defense against *A*. *fumigatus* infection, we performed another experiment in which we commenced the time-lapse acquisition at 24 HPI, using the dual fluorescent reporter line to follow macrophages and neutrophils. Acquisition continued over a time period of 16 hours (Supplementary Movie SM5). In our interpretation this captures several macrophages, heavily laden with *A*. *fumigatus* conidia, which germinate and grow hyphae from within the macrophages after phagocytosis. Taken together, these observations point to a fundamentally different mode of pathological development in the two *Aspergillus* species. Whereas *A*. *niger* appears dependent on evading the innate immune cells long enough to germinate and grow hyphae, *A*. *fumigatus* appears to be able to withstand phagocytosis and to grow hyphae, which will eventually burst through the phagocyte. This is in good agreement with the results from the survival rate experiments, indicating that *A*. *niger* causes very little mortality unless it is delivered at a dose that can overwhelm the immune response.

### Galactofuranose-deficient mutants of *Aspergillus niger* and *Aspergillus fumigatus* show attenuated virulence, but still elicit species-specific innate immune responses

**Figure 5:**
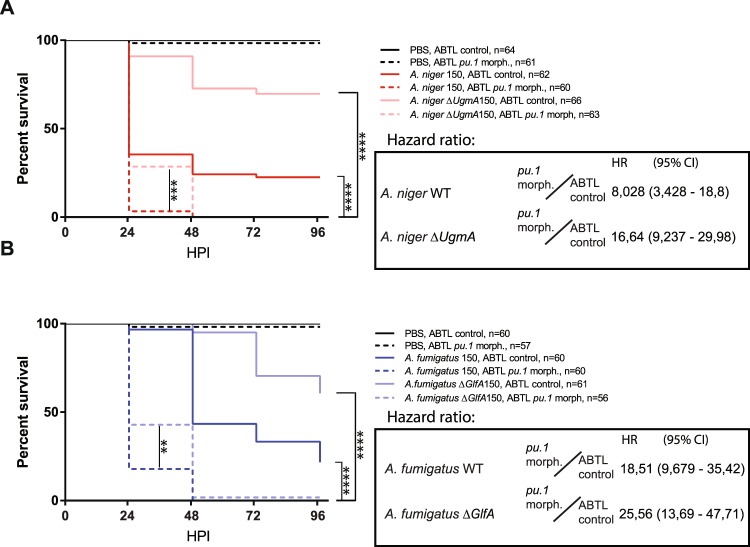
Survival rate analysis reveal significantly attenuated pathogenicity of galactofuranose deficient *Aspergillus* mutant strains. Survival of control (no morpholino) and immunodeficient *pu*.*1* morphant zebrafish embryos infected with 150 conidia of WT and gal*f* deficient mutant strains *A*. *niger* or (**A**) and *A*. *fumigatus* (**B**) at 28 HPF, survival was monitored at 24, 48, 72 and 96 hours post infection (HPI). Each graph display combined data from three independent biological replicates. Statistical curve comparisons by Mantel-Cox test, **p ≤ 0,01; ***p ≤ 0,001 ****p ≤ 0,0001. Hazard ratio calculations by Mantel-Haenszel method.

Galactofuranose, a component of the cell wall of all *Aspergillus* species, has been shown to be present at all early stages of the lifecycle, including dormant conidia, germinating conidia and growing hyphae of *A*. *fumigatus*^31,32^. Having characterized the different infectious behavior of *A*. *niger* and *A*. *fumigatus*, we wished to investigate whether the differences in interactions with the immune system would influence the properties of Gal*f* as a virulence factor. To approach this question, we compared the pathogenicity of WT *A*. *niger* and *A*. *fumigatus* to that of their respective Gal*f* deficient mutants (*A*. *niger ΔugmA* and *A*. *fumigatus ΔglfA*) in control and *pu*.*1* knockdown embryos (Fig. 5). The result clearly shows that Gal*f*-deficient strains of both *Aspergillus* species exhibit attenuated pathogenicity. As was the case with the WT strains, protection from infection and pathogenicity of the Gal*f-*deficient strains in both species was clearly dependent on a functioning innate immune system, as immunocompromised *pu*.*1* morphants, exhibited drastically increased mortality, compared to the immunocompetent controls (Fig. 5). Following the immune cell migratory responses by time lapse confocal microscopy the Gal*f* mutants appear to elicit similar innate immune responses to their respective WT strains (Supplementary Movies SM6–9). Leukocyte quantification revealed similar initial inflammatory responses in terms of leukocyte migration in response to the mutants both at 7 and 24 HPI compared to their respective parental strains (Supplemental Fig. 1). Thus, it does not appear that the attenuated pathogenicity of the Gal*f* synthesis mutants is directly related to drastically different interactions with immune cells.

### Decreased virulence of galactofuranose synthesis mutants correlates with slower hyphal growth

**Figure 6:**
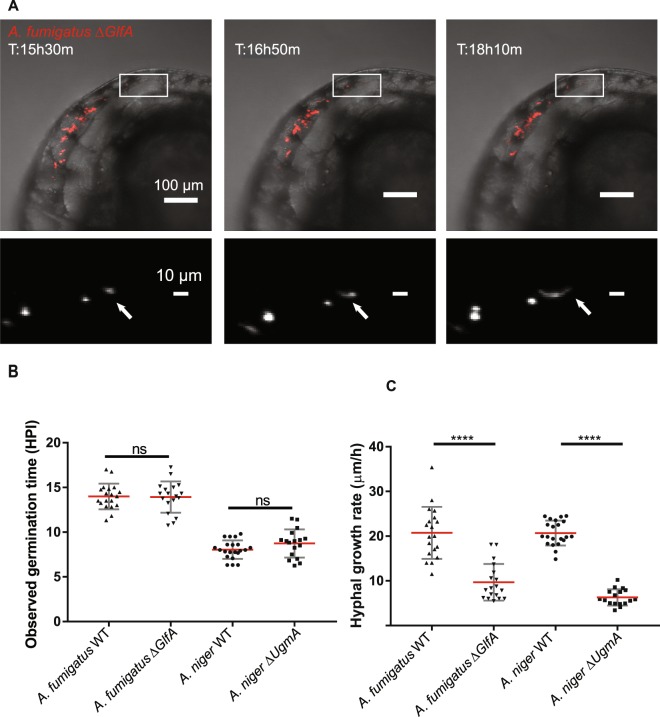
Timelapse microscopy based *in vivo* analysis of germination and growth rate phenotypes in galctofuranose deficient *Aspergillus* mutant strains. (**A**) Example of time-lapse microscopic assessment of hyphal growth-rate after injection of 150 conidia of the *A*. *fumigatus ∆glfA* mutant, in a *pu*.*1* morphant embryo. Upper panels show bright field images overlaid with the red fluorescent channel. The lower panel shows a blow-up of a specific area, marked in the upper panels, from the red fluorescent channel with signal in white. Individual confocal stacks of parallel time lapse acquisitions were analyzed to estimate hyphal growth rates. (See also Supplementary Movies SM10–13). (**B**) Analysis of germination time in confocal stacks of time-lapse microscopy experiment comparing *A*. *fumigatus* and *A*. *niger* WT to their respective Gal*f* deficient mutant strains at an infectious burden of approximately 150 conidia. No statistically significant difference could be detected between WT and gal*f* deficient strains in either species. (**C**) Analysis of approximate hyphal growth rate from confocal stacks. Plots derived from 17–21 independent hyphal growth measurements in 7–8 different *pu*.*1* morphant embryos from three independent biological replications each. The galactofuranose deficient mutants of both *Aspergillus* species exhibit significantly reduced rates of hyphal elongation *in vivo*. (**B**,**C**) Each scatterplot depicts the 17–21 data points generated from 7–8 individual *pu*.*1* morphant embryos covering three biological replications of each species and genotype. ****p ≤ 0,0001 by unpaired t test with Welsh correction.

While investigating the pathogenicity of the mutant fungal strains it was interesting to note that while the Gal*f-*deficient mutant strains of both *Aspergillus* species, like WT strains, effected nearly complete mortality in *pu*.*1* morphants, they did so slightly more slowly than the WT strains at the same infectious burdens (Fig. 5A,B). Though the survival of embryos to the Gal*f* deficient fungal strains was clearly dependent on cells of the innate immune system, the slight temporal delay of mortality of these fungal strains even in the absence of a functional innate immune system, indicates that elements inherent to the fungal physiology and growth are contributing to their loss of virulence. Several explanations could be offered to account for this, but most likely the attenuated pathogenicity of the Gal*f*-deficient *Aspergillus* strains might be caused by a decreased germination rate or hyphal growth rate. *In vitro* growth rate assessment did not reveal any major attenuation of growth rate of the Gal*f* deficient strains (Supplemental Fig. 2). Aiming to investigate this in the most appropriate environment, i.e. in an infected subject but without the growth-attenuating effects of immune cells, we analyzed confocal time-lapse videos which were acquired in parallel, to follow the germination and growth of WT and Gal*f-*deficient mutant conidia of *A*. *niger* and *A*. *fumigatus*, in *pu*.*1* morphant embryos. For each species and genotype we analyzed the time of germination based on the first observed germination events in *pu*.*1* morphant embryos captured by confocal time-lapse microscopy (Fig. 6A,B). The results of these experiments revealed no significant difference in the germination time between the gal*f* deficient mutant and parental strains of either species. With an average germination time of approximately 14 hours *A*. *fumigatus* appeared to take longer to germinate in the host environment compared to *A*. *niger* which exhibited an average germination time of 8 hours. Analyzing the increase in hyphal length between frames in the confocal time lapse microscopy series we could quantify an approximate rate of hyphal growth. The gal*f* deficient mutant strains of both species exhibited drastically decreased hyphal growth rates compared to WT (Fig. 6C and Supplementary Movies SM10–13). We propose this attenuated hyphal growth rate of the Gal*f*-deficient mutant strains as the most probable cause of their decreased pathogenicity.

## Discussion

*A*. *fumigatus* is well known for causing more serious infections in human patients than other fungi belonging to the *Aspergillus* genus, but the mechanisms underlying this higher pathogenicity are poorly understood. Here we used a well-established zebrafish hindbrain infection model to study differences in virulence between *A*. *fumigatus* and *A*. *niger*, focusing on the interaction of these fungi with the host innate immune cells, macrophages and neutrophils. The model is suitable for simple yet detailed non-invasive *in vivo* analysis of infectious progression based on the morphological alterations of the *Aspergillus* early lifecycle in dynamic interactions with innate immune cells. Performing parallel experiments with *A*. *fumigatus* and *A*. *niger* allowed us to compare and contrast these two species in terms of their infectious behavior and point out fundamental differences between the two species of *Aspergillus*. This led us to propose an explanation for the drastically higher incidence of infection caused by *A*. *fumigatus* compared to other species of *Aspergillus*.

**Figure 7:**
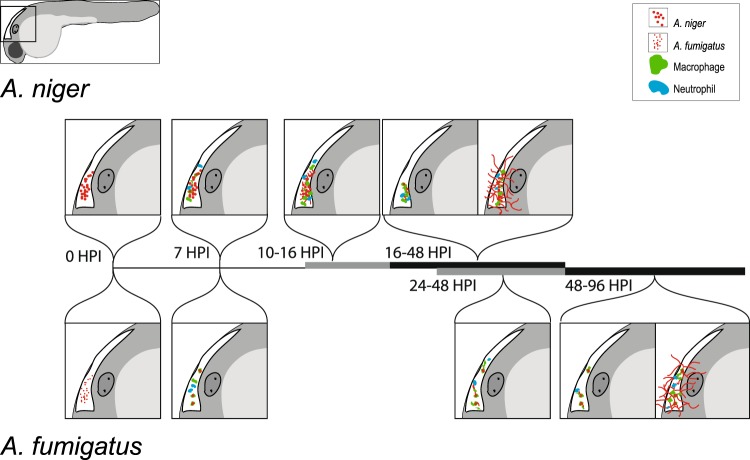
Comparative model of *A*. *niger* and *A*. *fumigatus* infectious development in the zebrafish hindbrain infection model. Events and approximate timing of infectious development in zebrafish embryos infected with *A*. *niger* in the upper half and *A*. *fumigatus* in the lower half of the model. *A*. *niger* conidia are depicted with larger red dots, to reflect their larger physical size relative to *A*. *fumigatus* conidia (*A*. *niger* conidia measures 4–5 µm in diameter versus 2–3 µm for *A*. *fumigatus*). *A*. *niger* elicits a robust migratory response from macrophages and neutrophils in the initial stages of infection. This leads to a strong innate immune cell presence before the conidia germinate, around 7–10 hours post infection (HPI). Despite the strong immune cell migration, phagocytosis is in many cases incomplete and extracellular conidia progress from dormancy to germination and hyphal growth, often between 10 and 16 HPI. Embryonic survival appears dependent on the immune system’s ability to curb the germination and hyphal growth, and as such the success of *A*. *niger* to cause infection is very dependent on the magnitude of the infectious burden, and very sensitive to mutations that impede hyphal growth rates such as the Δ*ugmA*. *A*. *fumigatus*, depicted in the lower half of the figure, causes a very similar initial immune cell migratory response. Conidia are very efficiently phagocytized, leading to the formation of large discrete clusters of phagocytized, but viable conidia inside macrophages. This development takes place within the initial hours after infection and is most often complete by 7 HPI. At later stages, starting from approximately 24 HPI, germinating conidia start to grow hyphae from within phagocytosing macrophages, while other macrophages can be observed to swarm around the growing hyphae, but seemingly unable to stop the growth.

We have gathered our observations in one comprehensive model of the development of infection of the two species of *Aspergillus* (Fig. 7). It is plausible that the increased incidence of infection caused by *A*. *fumigatus* is, at least partially, related to the ability of this species to withstand, germinate and grow hyphae after phagocytosis by macrophages. This behavior we have not observed for *A*. *niger*. Infection by *A niger* would therefore be expected to occur only in the cases when conidia are allowed to germinate and grow hyphae unchallenged by the immune cells. This notion is supported by the observation that the rate of *A*. *niger-*induced mortality drops rapidly to near zero when the infectious burden is reduced to 50 conidia, while *A*. *fumigatus* maintains significant mortality at this reduced challenge dose (Fig. 1C). Thus it would appear that *A*. *niger* primarily causes progressive infection and mortality when it is applied in a sufficiently high dose to overwhelm the initial immune response, whereas *A*. *fumigatus* is able to cause a fatal infection in spite of an adequate initial macrophage response. Importantly, the lower infectious dosages where *A*. *niger* was entirely contained are likely to be closer to those a human might encounter. The primary objective of our experiments applying higher dosages, is to force an infection to occur even with the less pathogenic *A*. *niger* which allows us to use microscopy based approaches to contrast the infectious route taken, and outline aspects of *A*. *fumigatus* infectious development that are likely to be critical to its enhanced pathogenicity.

Our observations regarding immune cell interactions of *A*. *niger* are difficult to discuss in a broader context as, to the best of our knowledge, no similar *in vivo* studies have explored *A*. *niger* infectious behavior of interactions with innate immune cells. *A*. *fumigatus*, as the more severe pathogen, has received substantial scientific attention. Our observations regarding the pathogenicity of *A*. *fumigatus* our observations indicate a more severe, and faster development of disease and mortality, than was reported in the study of Knox and coworkers^22^ and as such are more in line with those reported by Herbst and coworkers^33^. The causes of these inter-lab differences are not clear but may relate to the genetic background of the WT zebrafish lines used. Whatever the underlying cause may be, one advantage of the higher observed speed of infectious development in *A*. *fumigatus* in our studies compared to the Knox paper^22^ is that we can more easily capture some of the pivotal events in the progression of infection. This has allowed us to definitively show that *A*. *fumigatus* can germinate and grow from inside macrophages after phagocytosis *in vivo* (Fig. 4E), an event which the authors of the Knox paper remarked that they had been unable to capture. The notion that the progress of *A*. *fumigatus* infection will in some cases be initiated from within phagocytizing macrophages has been inferred from *in vitro* studies^4^, but to our knowledge this is the first time germination and hyphal growth from within phagocytizing macrophages has been demonstrated directly *in vivo*.

In the light of the observed differences between the two *Aspergillus* species of this study, as well as *in vitro* comparative studies comparing *A*. *fumigatus* to *A*. *terreus*^4^, it seems that the infectious behavior of *Aspergillus* may in many cases be fundamentally different across species. Therefore, it is very interesting to note that deficiency in the synthesis of the same cell wall component, galactofuranose (Gal*f*), affects both species in a similar manner, decelerating hyphal growth and significantly attenuating pathogenicity. We believe the Gal*f* synthesis pathway deserves further attention as a potential candidate for broad ranging *Aspergillus* medical intervention. The zebrafish hindbrain infection model, with its demonstrated applicability for investigations of immune cell infiltration behavior as well as a means to study the behavior of the fungal infectant *in vivo*, will be a valuable addition to further research efforts to resolve the relative importance of different cell wall components to overall infectious behavior of *Aspergillus* species. The demonstration of the *A*. *fumigatus* infectious route through the macrophage has now been observed by several independent studies apart from ours^22,33,34^. Further investigations into the relevant intracellular fungal derived signaling as well as relief strategies such as the lateral macrophage to macrophage transfer of conidia^34^ are likely to contribute further possible means of intervening in the infectious development of *A*. *fumigatus*.

## Experimental Procedures

### Zebrafish husbandry

Wild-type AB/TL, and transgenic Tg(*mpeg1*:*eGFP*^*gl22*^)^35^, Tg(*mpeg1*:*mCherryF*^*ump2*^)^30^ and Tg(*mpx*:*GFP*^*i114*^)^29^ zebrafish lines, adults and embryos, were handled in compliance with the local animal welfare regulations and maintained according to standard protocols (zfin.org). Breeding of zebrafish adults was approved by the local animal welfare committee (DEC) of the University of the Leiden, under license number 10612 and in compliance with international guidelines specified by the EU Animal Protective Directive 2010/63/EU. All studies in this work was performed on embryos/larvae before the free feeding stage, no adult fish were sacrificed, and experiments did not fall under animal experimentation law according to the EU Animal Protection Directive 2010/63/EU. Adult zebrafish were kept at 28 °C in an aquarium system with light day/night cycle of 14/10 hours, respectively. Embryos were cultured at 28.5 °C in egg water (60 μg/ml sea salt, Seramarin, Heinsberg, Germany). Prior to fungal microinjections or microscopic imaging embryos were anesthetized in egg water medium containing 0.02% (w/v) buffered Tricaine (3-aminobenzoic acid ethyl ester; Sigma-Aldrich, St Louis, MO, USA).

### Morpholino knock-down

Morpholino oligomer targeting *pu*.*1*^28^ was obtained from Gene Tools. 1 nl of morpholino solution at 1 mM was injected into the central region of the yolk of zebrafish embryos at the 1–2 cell developmental stage.

### Fungal conidia isolation and inoculum preparation

In this study the following fungal strains were used: *A*. *fumigatus* D141^36^, *A*. *niger* N402^37^ wild-types and their isogenic cell wall mutants *A*. *fumigatus ∆glfA*^9^ and *A*. *niger ∆ugmA*^8^. Fungi were grown on solid complete medium (CM) containing 1% (w/v) glucose, 7 mM KCl, 11 mM KH_2_PO_4_, 70 mM NaNO_3_, 2 mM MgSO_4_, 76 nM ZnSO_4_, 178 nM H_3_BO_3_, 25 nM MnCl_2_, 18 nM FeSO_4_, 7.1 nM CoCl_2_, 6.4 nM CuSO_4_, 6.2 nM Na_2_MoO_4_, 174 nM EDTA, 0.5% (w/v) yeast extract and 0.1% (w/v) casamino acids for 72 h at 37 °C for A. fumigatus and at 30 °C for A. niger. For the cell wall mutants 1 M sorbitol was added to CM. After 72 hours of growth, spores were harvested with 10 ml of PZ (0.9% (w/v) NaCl) and filtered through 2 layers of sterile Mira cloth. Spore inoculum concentration was measured by automated cell counter (Bio-Rad TC20) and adjusted to final concentration of 1.5 × 10^8^ spores/ml in 2% (w/v) polyvinylpyrrolidone (PVP) in phosphate-buffered saline (PBS). The obtained spore suspensions were used within 4 weeks.

Fluorescent labeled *A*. *niger* and *A*. *fumigatus* strains were constructed by co-transformation of plasmid pgpdA-mCherry with plasmid pAN7.1^38^. Hygromycin resistant transformants were purified twice and analyzed by fluorescence microscopy to identify transformants in which the FP construct was integrated into the genome. Transformation of *A*. *niger* and *A*. *fumigatus* respectively were performed as described previously^39^. Table 1 provides an overview of the strains utilized in this study.

**Table 1:** Overview of *Aspergillus* strains.

Species	Strain	Description	Reference
*A*. *niger*	N402	cspA1 (WT)	^37^
	MA87.6	ugmA::pyrG in MA70.15	^8^
	MA296.1	PgpdA-dsRed + pAN7.1 in N402	This study
	MB2.1	ugmA::nicB in MA323.1	^13^
	MB8.1	PgpdA-dsRed + pAB4.1 in MB2.1	This study
*A*. *fumigatus*	D141		^36^
	*∆glfA*	glfA::phleo in D141	^9^
	EL4.1	PgpdA-dsRed + pAN7.1 in D141	This study
	EL8.1	PgpdA-dsRed + pAN7.1 in ∆glfA	This study

Conidia inocula were adjusted to a final concentration of 1.0 × 10^8^ conidia/ml, as previously described^22^. Injection dosages were verified microscopically by injection on a plate during zebrafish injections, whereby conidial number can be conveniently counted at 40 times magnification. Unless otherwise stated 150 conidia were injected into the hindbrain ventricle via the otic vesicle zebrafish embryos at 30 hours post fertilization (HPF), as previously described^40^. For survival assays embryos were monitored every 24 h until 5 DPF.

### Microscopy

Infected embryos were anesthetized and embedded in 1% (w/v) low-melting-point agarose (Hispanagar) on a 40 mm glass bottom dish. Timelapse confocal stacks were acquired using a laser-scanning confocal microscope Nikon Eclipse Ti using 20x/0.75 objective for time-lapse imaging. For imaging of fixed embryos a LeicaTCS SPE with 20x/0.70 and 63x/1.20 objectives (Leica Microsystems) confocal microscope was used.

### Statistical analyses

Survival experiments were evaluated using the Kaplan-Meier method. Pairwise comparisons of survival curves were performed using the Log-rank (Mantel-Cox) test. Analysis was done using GraphPad Prism version 6.0. Statistical significance was assumed at p-value below 0.05.

## Supplementary information

supplementary material

SM1 Timelapse movie of macrophage responses to A. niger from 4-16 hours post infection

SM2 Timelapse movie of neutrophil responses to A. niger from 4-18 hours post infection

SM3 Timelapse movie of macrophage responses to A. fumigatus 4-16 hours post infection

SM4 Timelapse movie of neutrophil responses to A. fumigatus from 4-24 hours post infection

SM5 Timelapse movie of macrophage and neutrophil responses to A. fumigatus from 24-40 hours post infection

SM6 Timelapse movie of macrophage responses to A. niger ΔugmA from 4-10 hours post infection

SM7 Timelapse movie of neutrophil responses to A. niger ΔugmA from 4-18 hours post infection

SM8 Timelapse movie of macrophage responses to A. fumigatus ∆glfA from 4-10 hours post infection

SM9 Timelapse movie of neutrophil responses to A. fumigatus ∆glfA from 4-24 hours post infection

SM10 Timelapse movie of A. niger WT germination and growth in a Pu.1 morphant zebrafish embryo from 4-20 hours post infection

SM11 Timelapse movie of A. niger ΔugmA germination and growth in a Pu.1 morphant zebrafish embryo from 4-20 hours post infection

SM12 Timelapse movie of A. fumigatus WT germination and growth in a Pu.1 morphant zebrafish embryo from 4-20 hours post infection

SM13 Timelapse movie of A. fumigatus ∆glfA germination and growth in a Pu.1 morphant zebrafish embryo from 4-20 hours post infection

SM14 Timelapse movie of macrophage responses to A. niger from 7-16 hours post infection

SM15 Timelapse movie of macrophage responses to A. niger from 7-19 hours post infection

## Data Availability

Data and fungal strains are available from the corresponding authors upon request.
