# Supplementary material for: Aspergillus fumigatus establishes infection in zebrafish by germination of phagocytized conidia, while Aspergillus niger relies on extracellular germination
Source: Sci Rep. 2019 Sep 5;9:12791. doi: 10.1038/s41598-019-49284-w (PMC6728357; doi:10.1038/s41598-019-49284-w)

Aspergillus fumigatus establishes infection in zebrafish by germination of phagocytized conidia, while *Aspergillus niger* relies on extracellular germination

Bjørn E. V. Koch[1], Nathalia H. Hajdamowicz[1], Ellen Lagendijk[1], Arthur F. J. Ram[1]*, Annemarie H. Meijer[1]*

[1]Institute of Biology Leiden, Leiden University, the Netherlands

*To whom correspondence should be addressed: a.f.j.ram@biology.leidenuniv.nl, a.h.meijer@biology.leidenuniv.nl

Supplemental material

Supplemental figure 1 Quantification of inflammatory responses to hindbrain injections of conidia

Quantification of macrophage **(A** and **C)** and neutrophil **(B** and **D)** migration in the hindbrain of embryos injected with *A. fumigatus, A. niger* or the galactofuranose deficient cellwall mutant strains *A. fumigatus* ∆*glfA, A. niger* ∆*ugmA*, live or heat-killed (HK) conidia, compared to control injections (2 % PVP/PBS) at 7 HPI **(A-B)** and 24 HPI **(C-D)**. *p≤0,05; **p≤0,01; ***p≤0,001; ****p≤0,0001 by one-way ANOVA with Dunnett's multiple comparisons test, n=8-9 per group.

Supplemental figure 2 *In vitro* growth rate assessment

Growth rates of the *Aspergillus* strains used in this study were assessed at 28°C, across a variety of pH conditions and richness of media. The Δ*ugm*A mutant of *A. niger* grew and sporulated better at higer pH on complete media (CM). On minimal media (MM) *A. niger* Δ*ugm*A grew less, but could still grow radial hyphae, and appeared more affected in sporulation. Apart from these manifestations no obvious attenuation of growth could account for the reduced pathogenicity of the Gal*f* deficient strains compared to parental ones.

Supplemental timelapse movies

SM1 Timelapse movie of macrophage responses to *A. niger* from 4-16 hours post infection

Approximately 150 red fluorescent *A. niger* conidia injected in the mpeg:GFP zebrafish macrophage reporter line. Clear macrophage infiltration was evident, but germination and hyphal growth still occurred (indicated with arrow).

SM2 Timelapse movie of neutrophil responses to *A. niger* from 4-18 hours post infection

Approximately 150 red fluorescent *A. niger* conidia injected in the Mpx:GFP zebrafish neutrophil reporter line. Clear neutrophil infiltration was evident, but germination and hyphal growth still occurred (indicated with arrow). It was not clear from the time lapse video whether the neutrophils displayed any preference for any specific *A. niger* morphology i.e. conidia or hyphae.

SM3 Timelapse movie of macrophage responses to *A. fumigatus* 4-16 hours post infection

Approximately 150 red fluorescent *A. fumigatus* conidia injected in the Mpeg:GFP zebrafish macrophage reporter line. Clear macrophage infiltration and efficient phagocytosis occurred throughout the first hours after infection. Macrophages heavily laden with large numbers of phagocytized conidia formed discrete clusters surrounded by large numbers of other macrophages (indicated with arrows).

SM4 Timelapse movie of neutrophil responses to *A. fumigatus* from 4-24 hours post infection

Approximately 150 red fluorescent *A. fumigatus* conidia injected in the Mpx:GFP zebrafish macrophage reporter line. In the first several hours after infection very little neutrophil infiltration could be seen. *A. fumigatus* conidia could be seen to be moving around and gradually form large bright clusters, presumably due to the actions of macrophages not labelled in this experiment. Around 14 hours post infection a dramatic shift in the neutrophil behavior could be detected, manifested by the abrupt and rapid migration of large numbers of neutrophils into the hindbrain (arrows). It was not obvious what caused this change, but damage associated molecular patterns (DAMPs) possibly released from a distressed or lysed macrophage could be an explanation. Alternatively they could be reacting to an abrupt shift in fungal derived molecular patterns.

SM5 Timelapse movie of macrophage and neutrophil responses to *A. fumigatus* from 24-40 hours post infection

Approximately 100 red fluorescent *A. fumigatus* conidia injected in the Mpeg1:mCherry/Mpx:GFP zebrafish double reporter line. As the fungal infectant and the macrophages were labelled with the same fluorophore, the acquisition settings were adjusted so that the conidia and growing hyphae appeared white due to signal saturation while macrophages appeared faint red. At the initiation of the experiment 3 discrete clusters of macrophages with high numbers of conidia can be seen (arrows), reminiscent of the observations at the end of SM3. Several of these clusters started growing hyphae during the course of the timelapse experiment (arrowhead), and several macrophages could be seen to swarm around the growing hyphae. Only at the very end of the experiment could a solitary

neutrophil be seen to interact with the growing hyphae (arrow). Interestingly the neutrophil appeared to selectively interact with the growing tip of the hyphae.

SM6 Timelapse movie of macrophage responses to *A. niger ΔugmA* from 4-10 hours post infection

Approximately 150 red fluorescent *A. niger ΔugmA* conidia injected in the Mpeg:GFP zebrafish macrophage reporter line. Similarly to the WT (SM1) clear macrophage infiltration and interactions between conidia and macrophages were evident. No germination events were observed.

SM7 Timelapse movie of neutrophil responses to *A. niger ΔugmA* from 4-18 hours post infection

Approximately 150 red fluorescent *A. niger ΔugmA* conidia injected in the Mpx:GFP zebrafish neutrophil reporter line. Similarly to the WT (SM2) clear neutrophil infiltration interactions with the conidia were evident. No germination events were visible.

SM8 Timelapse movie of macrophage responses to *A. fumigatus ∆glfA* from 4-10 hours post infection

Approximately 150 red fluorescent *A. fumigatus ∆glfA* conidia injected in the Mpeg:GFP zebrafish macrophage reporter line. As in the WT, clear macrophage infiltration and efficient phagocytosis occurred throughout the first hours after infection.

SM9 Timelapse movie of neutrophil responses to *A. fumigatus* Δ*glfA* from 4-24 hours post infection

Approximately 150 red fluorescent *A. fumigatus* Δ*glfA* conidia injected in the Mpx:GFP zebrafish macrophage reporter line. Similarly to WT, very little neutrophil infiltration was evident in the first several hours after infection. Just as was the case in WT, *A. fumigatus* Δ*glfA* conidia could be seen to be moving around and gradually form large bright clusters, presumably due to the actions of macrophages not labelled in this experiment. The eventual neutrophil infiltration response, though slightly less dramatic and occurring slightly later, at approximately 18 HPI (arrows), resembled the behavior observed in WT (SF4).

SM10 Timelapse movie of *A. niger* WT germination and growth in a *Pu.1* morphant zebrafish embryo from 4-20 hours post infection

Approximately 150 red fluorescent *A. niger* WT conidia injected in the hindbrain of *Pu.1* morphant zebrafish embryos. Germination could be observed, commencing nearly simultaneously from many different conidia (arrows).

SM11 Timelapse movie of *A. niger ΔugmA* germination and growth in a *Pu.1* morphant zebrafish embryo from 4-20 hours post infection

Approximately 150 red fluorescent *A. niger ΔugmA* conidia injected in the hindbrain of *Pu.1* morphant zebrafish embryos. Germination appeared very similarly to in WT, but hyphal growth was less rapid than in WT (arrows).

SM12 Timelapse movie of *A. fumigatus* WT germination and growth in a *Pu.1* morphant zebrafish embryo from 4-20 hours post infection

Approximately 150 red fluorescent *A. fumigatus* WT conidia injected in the hindbrain of *Pu.1* morphant zebrafish embryos. Like *A. niger g*ermination occurred nearly simultaneously from many different conidia (arrows), but clearly later than in *A. niger.*

SM13 Timelapse movie of *A. fumigatus* ∆*glfA* germination and growth in a *Pu.1* morphant zebrafish embryo from 4-20 hours post infection

Approximately 150 red fluorescent *A. fumigatus* ∆*glfA* conidia injected in the hindbrain of *Pu.1* morphant zebrafish embryos. The timing of germination (arrows) was very similar to WT, but the elongation rate of growing hyphae was lower than in WT.

SM14 Timelapse movie of macrophage responses to *A. niger* from 7-16 hours post infection

Approximately 150 red fluorescent *A. niger* WT conidia injected in the hindbrain of mpeg:GFP zebrafish macrophage reporter line. Clear macrophage infiltration was evident, but numerous events of germination and hyphal growth still occurred.

SM15 Timelapse movie of macrophage responses to *A. niger* from 7-19 hours post infection

Approximately 150 red fluorescent *A. niger* WT conidia injected in the hindbrain of mpeg:GFP zebrafish macrophage reporter line. Clear macrophage infiltration was evident, but numerous events of germination and drastic hyphal growth still occurred.

Supplemental figure 1

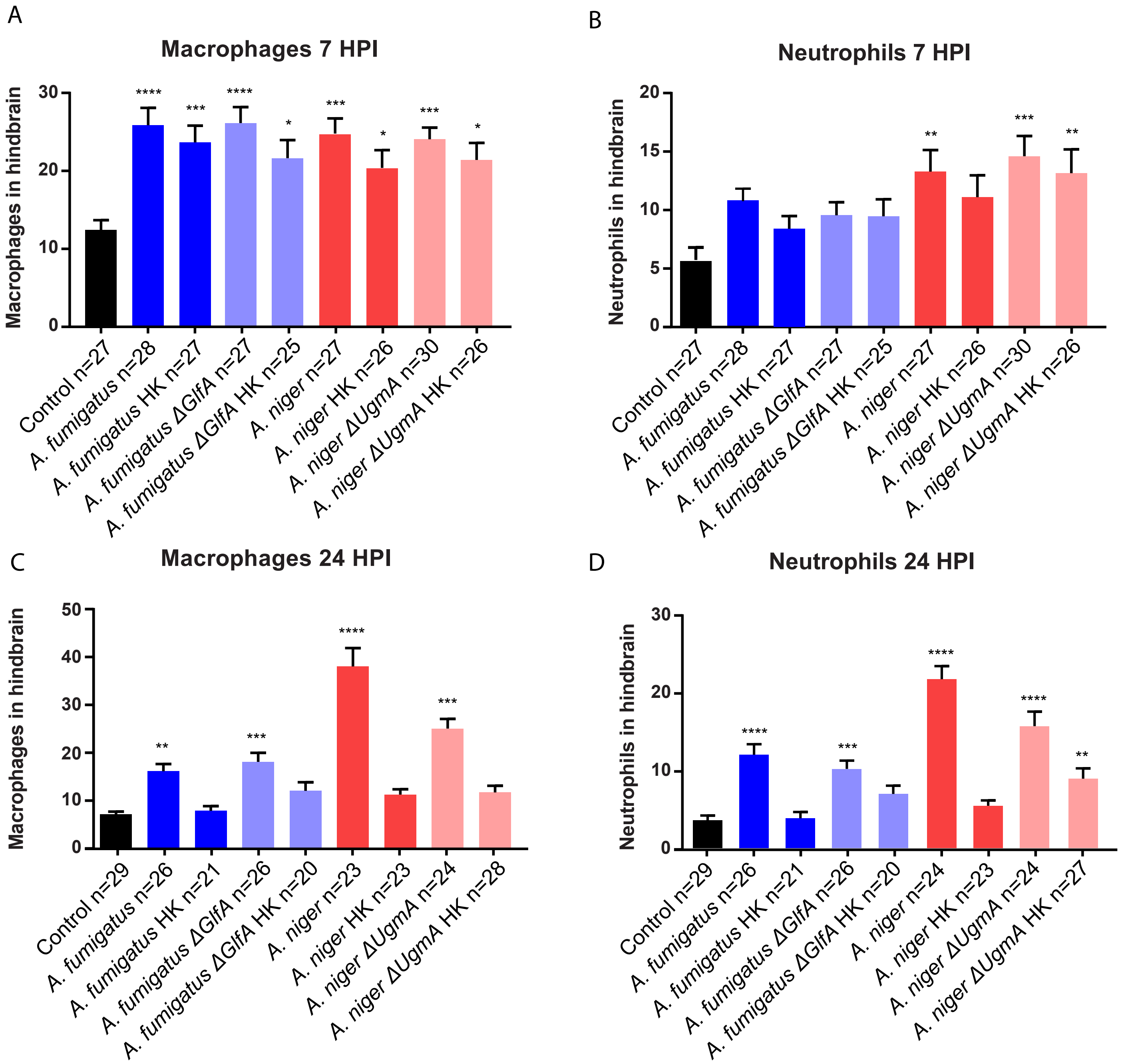

Supplemental figure 2

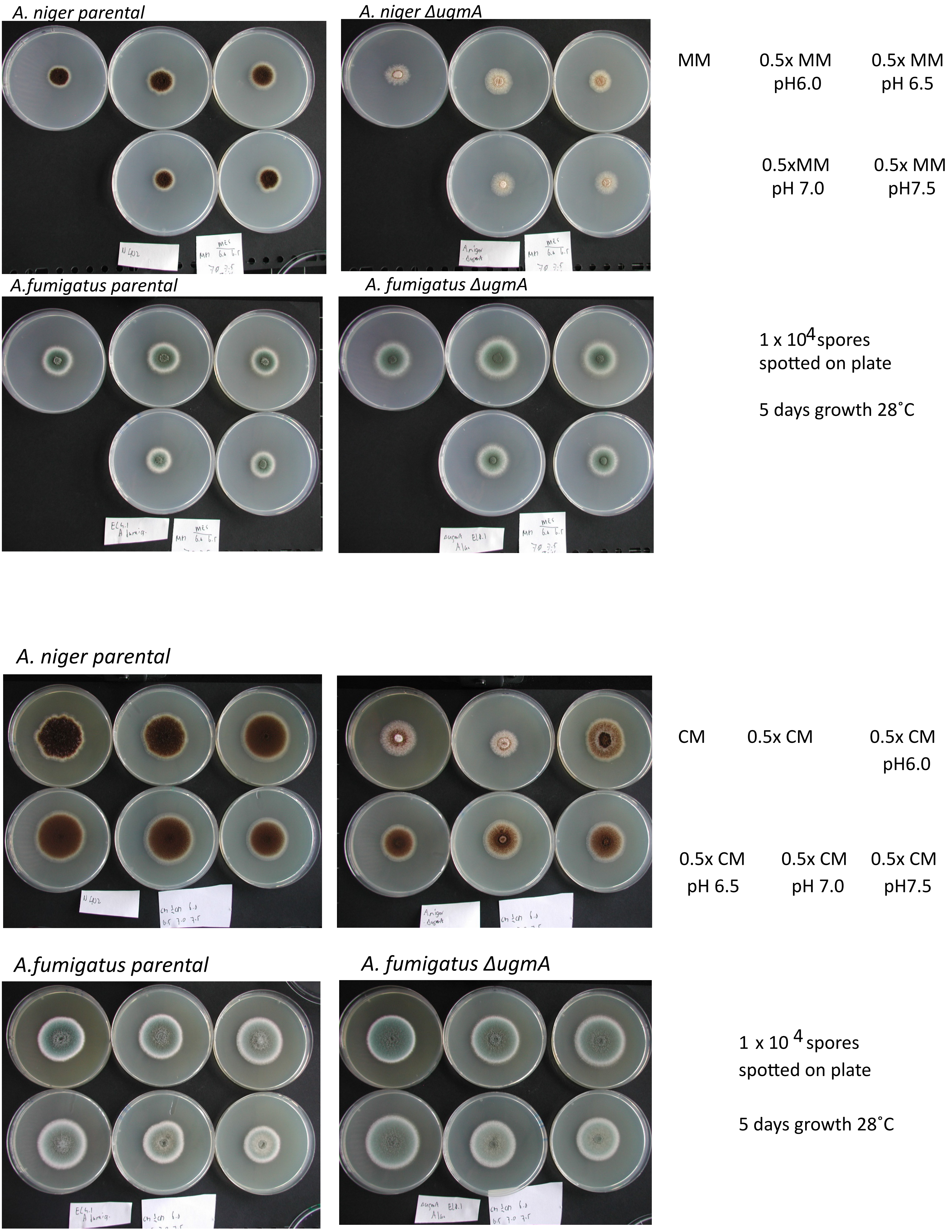

Supplement: Supplementary file 1 — supplementary material [file 41598_2019_49284_MOESM1_ESM.pdf]
